# Case Report: Characteristic findings of jugular foramen chordoma on long echo-time fat-suppressed T2-weighted imaging

**DOI:** 10.3389/fonc.2026.1785970

**Published:** 2026-05-15

**Authors:** Kai Chen, Mowei Dou, Lijing Deng, Yi Lei

**Affiliations:** 1 Department of Medical Imaging, The Fourth People’s Hospital of Shenzhen (Shenzhen Samii Medical Center), Shenzhen, China; 2 Department of Neonatology, Shenzhen Third People’s Hospital (The Second Affiliated Hospital, Southern University of Science and Technology), Shenzhen, China

**Keywords:** chordoma, jugular foramen, long echo time, magnetic resonance imaging, T2-weighted imaging

## Abstract

Chordomas of the jugular foramen (JF) are rare and pose a diagnostic challenge, as their non-specific clinical and conventional imaging features often overlap with more common lesions such as schwannomas and paragangliomas. We present the case of a 41-year-old female with a JF chordoma. While routine MRI revealed a T2-hyperintense mass, the critical diagnostic clue was provided by long echo-time (long-TE) fat-suppressed T2-weighted imaging (T2WI, TE = 280ms), which uniquely depicted a distinctive “beehive-like” internal architecture. This report highlights the value of this sequence in effectively revealing the internal architecture of JF chordomas, serving as a crucial adjunct for accurate preoperative diagnosis, particularly for stroma-poor variants.

## Introduction

Chordomas are rare, low- to intermediate-grade malignant neoplasms arising from notochordal remnants, with a strong predilection for the axial skeleton (1). Origin in the jugular foramen (JF) is exceptionally rare. However, a comprehensive review (2) reported that, in a cohort of JF tumors (18 schwannomas, 6 meningiomas, 5 chordomas, and 1 metastatic carcinoma), chordomas accounted for approximately 16.7% of cases (5/30). The diagnostic challenge is significant; patients typically present with non-specific symptoms, such as tinnitus, hearing loss, and lower cranial nerve palsies, which overlap extensively with more common JF pathologies, such as schwannomas, paragangliomas, and meningiomas (3, 4). Conventional computed tomography (CT) and magnetic resonance imaging (MRI) often reveal non-specific features, such as bone erosion and T2-hyperintensity, which are insufficient for a definitive diagnosis (5).

The histological spectrum of chordoma includes conventional, chondroid, and dedifferentiated subtypes (1), with a further relevant classification distinguishing between stroma-rich and stroma-poor variants (6). This distinction has imaging correlates, as stroma-poor chordomas tend to exhibit markedly high signal intensity on T2WI, a characteristic shared by other common JF lesions, leading to diagnostic ambiguity (2). We present a case of a surgically and histologically confirmed JF chordoma in which the application of a long echo-time (long-TE) fat-suppressed T2-weighted imaging (T2WI) sequence was pivotal in revealing a characteristic “beehive-like” internal architecture, providing a decisive preoperative diagnostic clue. This report adheres to the CARE guidelines to highlight this unique imaging finding and its clinical significance.

## Clinical data

A 41-year-old female presented with a primary complaint of progressive right-sided tinnitus and hearing loss persisting for over one year. She also reported intermittent numbness on the right side of her face. Her medical history was unremarkable, with no history of genetic syndromes, head and neck irradiation, or similar familial conditions.

In early 2023, the patient suddenly developed tinnitus in the right ear. The volume was low, did not resolve with rest, and persisted during sleep. No further treatment was pursued at that time. In August 2023, a head MRI performed at an outside hospital indicated a space-occupying lesion in the right jugular foramen, suggestive of a schwannoma. No immediate further intervention was undertaken. In August 2024, the patient presented to our institution seeking further management. A comprehensive neurological examination revealed complete sensorineural hearing loss in the right ear. The remainder of the cranial nerve examination was within normal limits.

**Figure 1:**
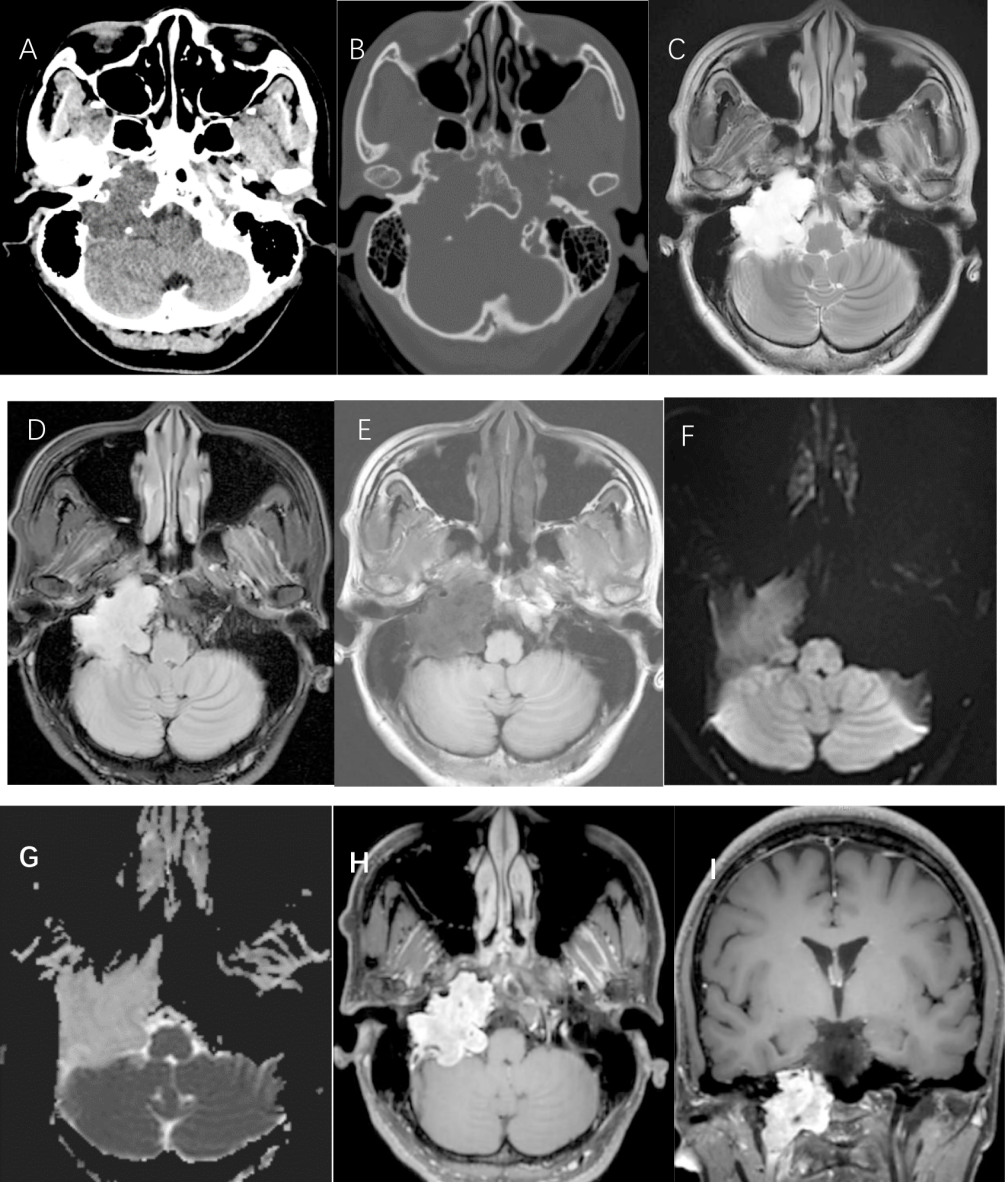
CT scans and MRI. **(A, B)** CT-scans. **(C-I)** MR images: **(C)** axial T2-weighted sequence; **(D)** axial T2-weighted fluid-attenuated inversion recovery sequence; **(E)** axial T1-weighted sequence; **(F)** diffusion-weighted imaging; **(G)** apparent diffusion coefficient imaging; and **(H, I)** T1-weighted gadolinium-enhanced sequence.

**Figure 2:**
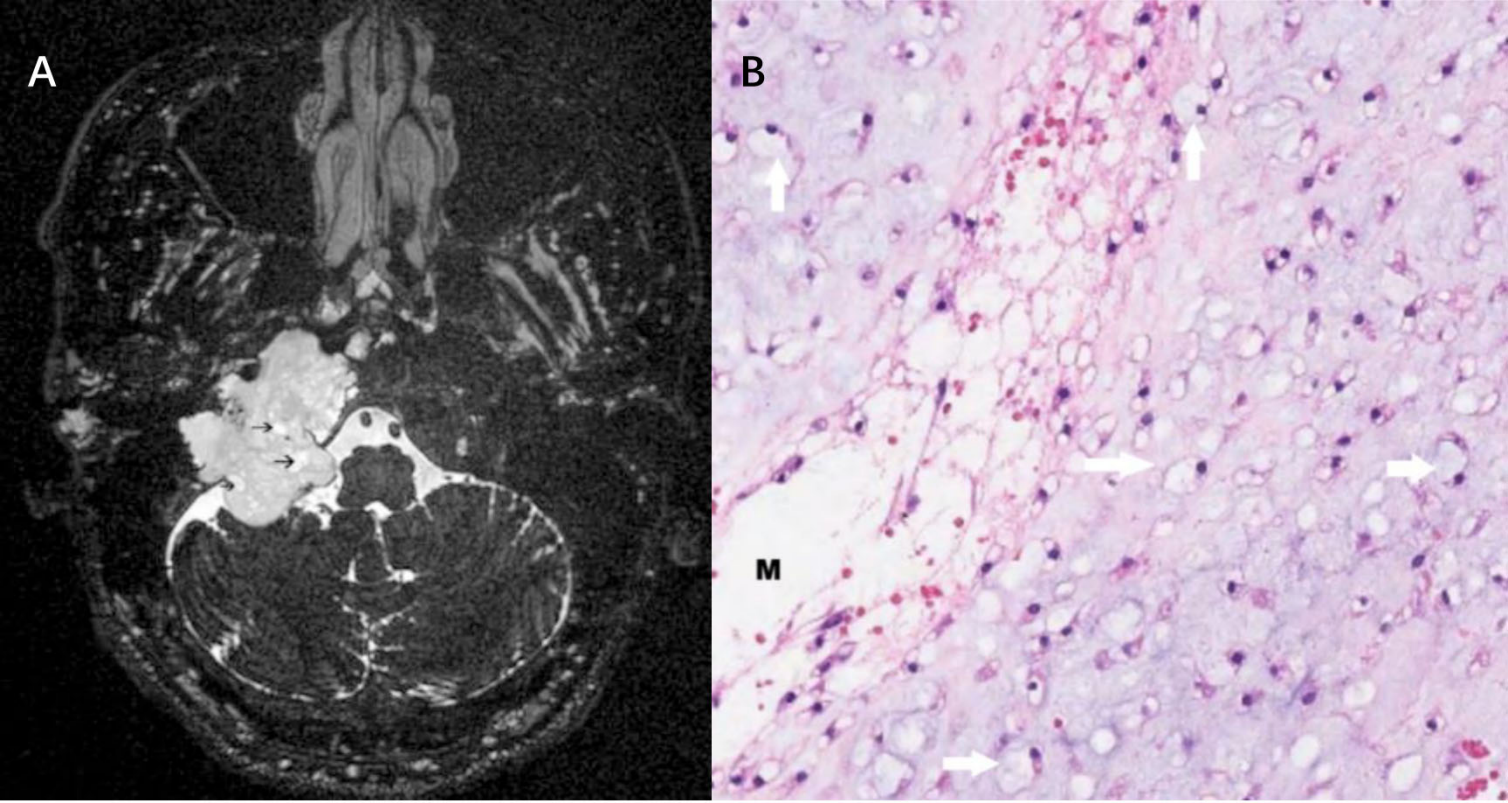
Long-TE fat-suppressed T2-weighted sequence andHE staining of the jugular foramen chordoma. **(A)** Long-TE fat-suppressed T2-weighted sequence showing the myxoid matrix or cystic components (black arrows). **(B)** HE staining (×27) of the chordoma showing physaliphorous cells (vacuolated cytoplasm, small nuclei; white arrows) in a chondroid stroma and extracellular mucin (M).

Following admission, the patient underwent imaging evaluation. Non-contrast CT demonstrated a lobulated soft-tissue mass in the right jugular foramen with associated bony erosion of the skull base (**Figures 1A, B**). Subsequent MRI revealed a mass that was hyperintense on T2-weighted imaging (T2WI), similar to cerebrospinal fluid (**Figure 1C**), and remained hyperintense on T2-FLAIR (**Figure 1D**). The mass was hypointense on T1-weighted imaging (T1WI) (**Figure 1E**) and showed no restricted diffusion on DWI/ADC sequences (**Figures 1F, G**). Post-contrast T1WI demonstrated marked heterogeneous enhancement (**Figures 1H, I**). The pivotal sequence — a long echo-time fat-suppressed T2WI acquired via a three-dimensional variable flip angle fast spin-echo (3D VFA-FSE) technique (repetition time [TR]/TE = 1300/280 ms, echo train length = 18, slice thickness = 0.4mm, field of view = 200×180, matrix = 368×368)—revealed scattered punctate and round hyperintensities within the tumor parenchyma, creating a distinctive “beehive-like” appearance (**Figure 2A**).

The patient underwent a subtotal resection of the tumor. Intraoperative findings confirmed a well-circumscribed tumor with a cystic-solid texture. Pathological examination of the resected specimen confirmed the diagnosis of conventional chordoma. It revealed characteristic clusters and cords of physaliferous cells (noted for their vacuolated cytoplasm) embedded within a myxoid matrix (**Figure 2B**). Immunohistochemistry was positive for S-100 and brachyury, which are definitive markers for chordoma.

The patient’s postoperative recovery was uneventful. At the 3-month follow-up, partial recovery of hearing was noted. Follow-up at 9 months post-surgery demonstrated further hearing improvement, and no new neurological deficits were observed, indicating a positive outcome following surgical intervention. Imaging revealed no increase in the size of the residual tumor at the right JF and no evidence of distant metastasis.

## Discussion

This case elucidates the significant diagnostic utility of long-TE fat-suppressed T2WI in the evaluation of JF chordomas. It differs from gradient-echo-based steady-state free precession (SSFP) sequences, such as FIESTA (GE) and 3D CISS (Siemens). SSFP sequences produce mixed T2/T1 contrast and are well suited for depicting fine anatomical structures, such as cranial nerves. In contrast, long-TE T2WI provides pure T2 weighting, making it more sensitive to fluid-rich or mucinous parts of the tumor. It may also be more effective than SSFP when identifying cystic-solid heterogeneity. Chordomas are composed of lobules of tumor cells (physaliphorous cells) within an abundant myxoid or chondroid matrix (7). The long-TE T2WI (280ms vs. a conventional 80ms) allows for greater signal decay from tissues with shorter T2 relaxation times, such as the cellular components of the tumor. In contrast, the myxoid matrix, with its intrinsically long T2 values, retains high signal intensity. This enhanced contrast vividly outlines the matrix-rich regions, creating the observed “beehive” or “honeycomb” pattern (5). The addition of fat suppression eliminates the high signal from surrounding marrow fat, further improving the conspicuity of this finding.

The differential diagnosis for a JF mass is broad (4), primarily including:

Jugular paraganglioma: typically demonstrates a “salt-and-pepper” appearance on T2WI and intense, early enhancement.

Schwannoma: often appears as a well-circumscribed, homogeneously T2-hyperintense mass, frequently with cystic components, but lacks the specific internal architecture seen in this case. The “beehive-like” hyperintensity observed in chordomas is distinct from the heterogeneous cystic-solid appearance typically seen in jugular foramen schwannomas (often with eccentric cysts) and the “salt-and-pepper” pattern characteristic of jugular paragangliomas (8).

Meningioma: usually isointense to gray matter on T2WI and may exhibit a dural tail sign.

Chondrosarcoma: shares many imaging similarities with chordoma, though some studies suggest lower apparent diffusion coefficient (ADC) values in chordomas may aid differentiation (3, 4). The relatively high ADC value (2.17×10^−3^ mm²/s) in this case is atypical for conventional chordomas. Moreover, ADC values are known to overlap among skull base tumors (9). Therefore, ADC should be interpreted alongside morphological features on long-TE fat-suppressed T2WI, rather than as a sole differentiating parameter.

While advanced techniques such as dynamic contrast-enhanced (DCE) MRI can provide additional parameters (e.g., K_trans_, Ve), these can be variable and scanner-dependent (10). The long-TE fat-suppressed T2WI sequence, however, is technically straightforward, highly reproducible across different MRI platforms, and adds minimal time to the standard protocol. It may also be useful for other skull base tumors; in schwannomas, the ‘target sign’ reflecting Antoni B tissue is more conspicuous on long-TE T2WI, which may aid differentiation from paragangliomas.

Our findings are limited to a single case of chordoma, a subtype with a mucin-rich matrix. Long-TE T2WI appearances may differ in conventional chordomas, which tend to have more cellular or calcified components, or in dedifferentiated chordomas. Whether this sequence is useful across chordoma subtypes remains unknown. Studies comparing long-TE T2WI findings with histopathological features across subtypes are needed.

## Conclusion

In conclusion, long-TE fat-suppressed T2WI is a simple yet highly effective sequence that can delineate the characteristic internal architecture of JF chordomas. The “beehive-like” sign provides a valuable and reproducible diagnostic clue, complementing routine MRI and aiding significantly in the differentiation from other jugular foramen lesions, particularly in challenging cases of stroma-poor chordoma. We advocate for the inclusion of this sequence in MRI protocols for evaluating complex skull base tumors.

## Data Availability

The raw data supporting the conclusions of this article will be made available by the authors, without undue reservation.
